# Humoral Immunity vs. *Salmonella*

**DOI:** 10.3389/fimmu.2019.03155

**Published:** 2020-01-21

**Authors:** Akiko Takaya, Tomoko Yamamoto, Koji Tokoyoda

**Affiliations:** ^1^Laboratory of Microbiology and Molecular Genetics, Graduate School of Pharmaceutical Sciences, Chiba University, Chiba, Japan; ^2^Department of Infectious Diseases, Medical Mycology Research Center, Chiba University, Chiba, Japan; ^3^Deutsches Rheuma-Forschungszentrum Berlin (DRFZ), A Leibniz Institute, Berlin, Germany

**Keywords:** humoral immunity, antibody, plasma cells, IgG, *Salmonella*

## Abstract

In primary infection with *Salmonella*, it has been reported—without consideration of *Salmonella*'s functions—that humoral immunity plays no role in the clearance of bacteria. In fact, *Salmonella* targets and suppresses several aspects of humoral immunity, including B cell lymphopoiesis, B cell activation, and IgG production. In particular, the suppression of IgG-secreting plasma cell maintenance allows the persistence of *Salmonella* in tissues. Therefore, the critical role(s) of humoral immunity in the response to *Salmonella* infection, especially at the late phase, should be re-investigated. The suppression of IgG plasma cell memory strongly hinders vaccine development against non-typhoidal *Salmonella* (NTS) because *Salmonella* can also reduce humoral immune memory against other bacteria and viruses, obtained from previous vaccination or infection. We propose a new vaccine against *Salmonella* that would not impair humoral immunity, and which could also be used as a treatment for antibody-dependent autoimmune diseases to deplete pathogenic long-lived plasma cells, by utilizing the *Salmonella*'s own suppression mechanism of humoral immunity.

## Introduction

The immune system, i.e., innate and adaptive immunity, can overcome many types of bacterial infections. The frontline against infection with bacteria such as *Salmonella* is innate immunity. *Salmonella* infection leads to enteric fever or diarrhea, often resulting in death of humans and animals. The pathogenesis of infection should be separately considered as two dynamics of the immune system vs. *Salmonella*: firstly, bacterial growth within 1 week after infection and, secondly, if protected from death, bacterial clearance after 1 week after infection. Early bacterial growth in mice is controlled by the Nramp gene, expressed in macrophages (1), and is suppressed by a T-cell-independent host response which requires granuloma formation and production of nitric oxide and cytokines such as tumor necrosis factor α (TNFα), interleukin 12 (IL-12) and interferon γ (IFNγ) (2–6). For clearance of the bacteria, innate immunity, namely the complement system and phagocytosis by macrophages, neutrophils and dendritic cells, are the most critical responses against the bacterial pathogens, while IFNγ and antibodies resulting from adaptive immunity also dramatically enhance the innate immune response. It has been thought that adaptive immunity itself dominantly works for secondary infection except for IFNγ from T cells. However, it remains enigmatic how adaptive immunity contributes to the clearance of *Salmonella* in the primary infection. We herein discuss the roles of humoral immunity against *Salmonella* for the clearance of the bacteria.

## Developing a Vaccine Against *Salmonella*

*Salmonella enterica* is a Gram-negative intracellular bacterium with over 2,500 different serovars identified until now. *Salmonella* Typhi (*S*. Typhi) and *S*. Paratyphi cause typhoid fever, a systemic febrile illness only affecting humans. The other numerous NTS serovars such as *S*. Typhimurium and *S*. Enteritidis infect many different hosts and results in diarrheal disease. NTS also causes severe, extra-intestinal, invasive bacteremia, referred to as invasive NTS (iNTS) disease (7). Immunocompromised individuals, including those infected with human immunodeficiency virus (HIV) or malaria, and infants are particularly at risk of acquiring iNTS disease (8–12). iNTS disease is estimated to cause 3.4 million cases of illness and 681,316 deaths annually, with 63.7% of all cases occurring in children under the age of five (8). Thus, infection with NTS is still a serious health concern. Moreover, the emergence of multidrug-resistant strains of *Salmonella* calls into question the future use of antibiotics to treat infection and further emphasizes the need for the development of the safer and more effective vaccines. While a vaccine against NTS is not currently available, it has been reported that naturally acquired antibodies against NTS reduce the risk of iNTS disease (13, 14). In contrast, infection with *S*. Typhi can be prevented by vaccination with attenuated strains, e.g., Ty21a. However, effective vaccines preventing iNTS disease are likely to differ from those protecting against *S*. Typhi infections. Furthermore, it is known that *Salmonella* generates chronic carriers; a chronic carrier state has been identified in 2.2% of patients with reported NTS, lasting from 30 days to 8.3 years (15). Although *Salmonella* invades myeloid cells and escapes phagocytosis, it is unclear why humoral immunity does not contribute to the clearance of *Salmonella* which continuously transfers among short-lived myeloid cells. Overall, the lack of a vaccine and the presence of chronic carriers suggests that NTS suppresses long-lasting humoral immunity, i.e., humoral memory.

## The Immune System vs. *Salmonella*

Infection of susceptible Nramp^−^ mice with *S*. Typhimurium provides a murine model for typhoid fever which bears many similarities to human *S*. Typhi infection. This *S*. Typhi model is ultimately fatal due to the inability of such mice to restrict bacterial growth *in vivo*. Administration of attenuated strains of *S*. Typhimurium as a model of vaccination resulted in the generation of immune memory against *Salmonella* and protection against death from challenge with a virulent strain of the bacteria (16, 17). The murine model infected with virulent *S*. Typhimurium showed similar pathogenesis on the early growth of bacteria. However, it seems unclear whether the model with attenuated *S*. Typhimurium really mimics the clearance of *Salmonella*, i.e., whether *S*. Typhi and *S*. Typhimurium are excluded from their hosts in a similar way. Many studies have discussed typhoidal disease using NTS strains based on the assumption that *S*. Typhi and *S*. Typhimurium utilize the same invasion system in the hosts. However, it is impossible to compare the mechanism on the clearance of *Salmonella in vivo*, because *S*. Typhi is not infectious in mice. If *S*. Typhi and *S*. Typhimurium are excluded by distinct bacterial clearances, the difference may affect the ability to generate vaccines against *S*. Typhi and *S*. Typhimurium.

Innate cells can have several roles to play during the early stage of an infection, including controlling bacterial replication and producing cytokines and chemokines that activate and recruit inflammatory cells to the site of infection. Macrophages, neutrophils and dendritic cells increase in number early after *Salmonella* infection and produce cytokines that are important for host survival, such as TNFα. All three phagocytic cell types also harbor bacteria during infection. IFNγ from natural killer cells at the very early infection phase and from T cells at the late infection phase can activate macrophages and promote phagocytosis (18). In addition to innate cells, the clearance of bacteria from the tissues also requires functional CD4 T cells (19), resulting in long-lasting specific immunity to re-challenge infection (20). Susceptible mice can resolve a primary infection with attenuated *Salmonella* strains which requires a functioning immune system that can develop a T-bet-dependent Th1 cell response and IFNγ production to activate infected macrophages (19, 21). Similarly, mice lacking IL-12, IFNγ, reactive oxygen species, or inducible nitric oxide, all have deficiencies in primary clearance of *Salmonella* (22, 23). In contrast, mice lacking B cells resolve primary infection with attenuated bacterial strains with similar kinetics to wildtype mice (24, 25), indicating that B-cell responses do not participate in the primary clearance of the bacteria. CD8 T cells are generally not thought to contribute to the primary clearance of attenuated *Salmonella*, based on studies using β2-microglobulin-deficient mice that lack class I-restricted CD8 T cells (19, 26). However, recent experiments in mice lacking classical MHC class Ia genes, perforin, or granzyme, show that CD8 T cells make a modest contribution to *Salmonella* clearance during the later stages of the primary response (27). Overall, these data suggest a primary role for CD4 Th1 cells, a modest role for CD8 T cells and no role for B cells in primary immunity to *Salmonella*. However, the roles of adaptive immunity were considered from the viewpoint of how the lymphocytes respond to the infection, without any consideration of how *Salmonella* may purposefully subvert the immune response for its own advantage.

## Humoral Immunity vs. *Salmonella*

Immunization and infection with *Salmonella* greatly affects hematopoiesis in a TNFα- and CXCL12-dependent manner (28, 29). *Salmonella* is known to activate myelopoiesis and suppress B lymphopoiesis (30). Interestingly, the disruption of B lymphopoiesis has been also reported on *Plasmodium* infection in mice (31), suggesting the similar mechanism to *Salmonella*. This dramatic change in cellular commitment/differentiation is very reasonable, because in the early phase of infection, the immune system requires as many innate cells as possible to fight against the infection. Expanded myeloid cells are able to kill a lot of *Salmonella*, but some become the host cells for *Salmonella* without phagocytosis. Furthermore, the provision of B cells to the periphery is impaired due to death of B cell precursors in the bone marrow (BM), resulting in an indirect advantage to *Salmonella* for their long-term persistence.

In general, antibodies can protect against bacteria mainly by facilitating the uptake of the pathogen by phagocytic cells, which then destroy the ingested bacteria. Antibodies do this in two ways: one is to coat the pathogen to be recognized by Fc receptors on phagocytic cells, which is called opsonization. Alternatively, antibodies binding to the surface of a pathogen can activate the proteins of the complement system. Complement activation results in opsonization of the pathogen by binding complement receptors on phagocytes. Other complement components recruit phagocytic cells to the site of infection, and the terminal components of complement can lyse certain microorganisms directly by forming pores in their membranes. Most intracellular pathogens spread by moving from cell to cell through the extracellular fluids. The extracellular spaces are protected by humoral immunity. Antibodies produced by plasma cells cause the destruction of extracellular microorganisms and therefore prevent the spread of intracellular infections. Phagocytes, *Salmonella*'s hosts, are short-lived and survive for 0.75 days (neutrophils, lifespan) (32), 18–20 h (phagocytic monocytes, half-life) (33), 1.5–2.9 days (dendritic cells, half-life) (34), and <7 days (peripheral macrophages, lifespan) (35). Therefore, in order to survive, *Salmonella* has to transfer into new host cells every 1–7 days passing through extracellular fluids containing antibodies. It is unknown how and why *Salmonella* can escape from antibodies in extracellular spaces when transferring into new host cells. In secondary immune responses, anti-S*almonella* IgG are critical for the enhancement of phagocytosis. However, anti-*Salmonella* IgG in the late phase of the primary immune response does not contribute to the clearance of the bacteria (23). This raises the following questions: what is the difference of anti-*Salmonella* antibodies in the primary and secondary immune responses? Is the affinity and/or amount of antibodies important? What other functions of *Salmonella* have to be also considered in the subversion of the immune response?

The activation of B cells and their differentiation into long-lived plasma cells is triggered by antigen and usually requires CD4 T cell help, presenting antigen on MHC class II. Bayer-Santos and his colleagues showed that a *Salmonella* protein, SteD depletes surface MHC class II and inhibits T cell activation (36). SteD localized in the Golgi network and vesicles containing the E3 ubiquitin ligase MARCH8 and MHC class II causing MARCH8-dependent ubiquitination and depletion of surface MHC class II and B7-2. A subset of effector CD4 T cells, known as T follicular helper cells, also control isotype switching and have a role in initiating somatic hypermutation of antibody variable V-region genes for affinity maturation mainly in germinal centers (GCs) of the spleen. Cunningham et al. indicated that GC formation is delayed when infected with *Salmonella* (37). However, GC-lacking CD40L (CD154)-deficient mice can normally induce the clearance of *Salmonella* in tissues. The formation of GCs and the affinity of antibodies do not affect the clearance of the bacteria. Di Niro et al. showed that *Salmonella* induces random activation, generating only a small fraction (0.5–2%) of *Salmonella*-specific plasma cells, and somatic hypermutation occurred efficiently at extrafollicular sites (38). Although it should be investigated how the abnormal induction consequently affects the immune responses, it is very intriguing why *Salmonella* does not allow immune cells to utilize the standard immune activation/maturation pathways. Following GC formation, B cells can differentiate into either short-lived plasma cells, memory B cells, or long-lived plasma cells. Memory B cells persist and are important for secondary immune responses against the same pathogen. Short-lived plasma cells temporally provide IgG, but do not survive for long periods of time. In contrast, long-lived plasma cells, or their precursors, migrate into the BM and persist in CXCL12-expressing stromal cells (39, 40). In general, IgG is the most critical antibody isotype for the clearance of bacteria and greatly contributes to the clearance of bacteria at least in the late phase of infection. In contrast, in the clearance of *Salmonella*, no role of B cells which has a potential to differentiate into IgG-secreting plasma cells has been reported. The distinction led to a possibility of *Salmonella*-specific suppression of humoral immunity, in particular IgG production as described below.

## *Salmonella* Attacks the Main Source of Igg

**Figure 1:**
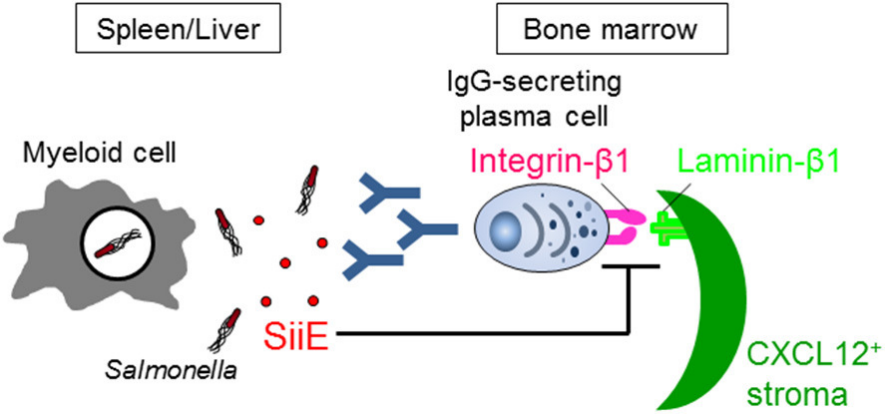
*Salmonella* SiiE suppresses the retention of IgG-secreting plasma cells in BM survival niches by competing with laminin β1. SiiE secreted by *Salmonella* competes with laminin β1 to interact with integrin β1. The competition induces the detachment and then deletion of IgG-secreting plasma cells from laminin β1^+^CXCL12^+^ survival niches of the BM.

McSorley and Jenkins showed (i) that *Salmonella* can similarly survive in the tissues of naive wild-type and B cell-deficient mice until day 35 after infection, suggesting that antibodies and B cells are not necessary for the clearance of *Salmonella*, and (ii) that injection of heat-killed *Salmonella* induces a provision of anti-*Salmonella* IgG from day 20, although data of anti-*Salmonella* IgG titers in mice infected with live *Salmonella* are lacking (24). However, if *Salmonella* actively suppresses B cell functions, the necessity of B cells for fighting the infection therefore fails to be evaluated by these studies. Very recently, we have shown that *Salmonella* inhibits the persistence of IgG-secreting plasma cells in the BM of mice, which are the main source of serum IgG, by secreting a *Salmonella* protein known as SiiE (41) (Figure 1). Mice infected with a SiiE-deficient strain markedly enhanced the provision of anti-*Salmonella* IgG and promoted the clearance of *Salmonella*, even in the primary infection. Given these results, the roles of antibodies and B/plasma cells therefore have to be re-evaluated.

SiiE is known as an adhesin, binding to carbohydrates in a lectin-like manner, thereby promoting attachment of *Salmonella* to polarized epithelial cells and enabling colonization (42, 43). SiiE is secreted by *Salmonella* and remains surface-associated during bacterial invasion (44). SiiE mediates the first direct contact to the host cell through binding to glycostructures containing N-acetyl-glucosamine and/or α2, 3-linked sialic acid (45). Recently, Li et al. suggested that MUC1, the transmembrane mucin that is highly expressed at mucosal surfaces including the stomach and the intestinal tract, is a receptor for SiiE that enables apical invasion into enterocytes (46).

SiiE is a large protein with a molecular weight of 595 kDa. It has 53 highly similar repeats of bacterial immunoglobulin (BIg) domains that determine the length and only short protein moieties of distinct structure at the very N- and C-terminal parts (43). The amino acid sequence from 129 to 168 in the short N-terminal moiety has high homology to murine laminin β1. The 236 amino acid residues in the short N-terminal moiety consist of eight heptad repeats with a coiled-coil structure that are flanked by regions with a predominantly β-sheet structure (43). The integrity of the coiled-coil structure is required for the proper retention of SiiE and thereby affects invasion of polarized cells, while the β-sheet domains appear to be essential for the control of release of SiiE. The central part of the coiled-coil structure, including amino acids 129–168, plays an especially essential role in the retention of SiiE (43). The homologous region in the C-terminal region of laminin β1 also has a coiled-coil structure, which is involved in the assembly of a laminin molecule (47). The C-terminal region also modulates the integrin binding affinities of laminins (48). We showed that SiiE can bind to integrin β1, a laminin receptor, on BM IgG-secreting plasma cells and competes with their adhesion to laminin (41). Only the SiiE-derived peptide which has high homology to murine laminin β1 was able to reduce the number of BM IgG-secreting plasma cells. Moreover, the attenuated SiiE-deficient *Salmonella* enhanced both the production of high titers of protective IgG against *Salmonella* and the memory response, suggesting that it may be a novel and efficient vaccine against *Salmonella*. Histological analyses of the BM revealed that IgG- but not IgM-secreting plasma cells bind to laminin β1. Thus, laminin β1^+^CXCL12^+^ stromal cells are an integral part of the survival niche for IgG-secreting plasma cells in the BM, a lesson learnt from *Salmonella*.

## Roles of Humoral Immunity Against *Salmonella* and New Generation of Vaccines

*Salmonella* SiiE reduces the number of BM IgG-secreting plasma cells (41). This reduction may have led to the underestimation of the roles of B cells, especially antibodies, in the late phase of the primary infection with *Salmonella*. If IgG production is not suppressed by *Salmonella* SiiE, humoral immunity, in particular IgG, is required for the clearance of *Salmonella* in the late phase of the primary infection (41). Infection with SiiE-deficient strain into B cell-deficient and wildtype mice should be examined in order to determine the precise role of humoral immunity in the late phase of primary infection with *Salmonella*. Since vaccines against NTS are not yet available, SiiE-deficient *Salmonella* may be the first efficient vaccine against NTS. It still remains unclear why vaccines against *S*. Typhi, but not NTS are available. Intriguingly, the *siiE* gene in *S*. Typhi has been reported as two distinct ORFs, suggesting that it is a pseudogene (49). The presence of a functional SiiE gene may be a reason for the differences in availability of vaccines against the two strains of *Salmonella*. Furthermore, SiiE impairs the persistence of all IgG-secreting plasma cells in an antigen-specific independent manner. This non-specific depletion of IgG-secreting plasma cells may result in the loss of long-lived plasma cells secreting IgG against many kinds of bacteria and viruses generated by previous vaccination or infection. Therefore, generating vaccines against NTS may be essential to avoid such a loss of vital of humoral memory. Other pathogens may also have an ability to suppress humoral immunity. Recent studies indicated that respiratory syncytial virus (RSV) infection fails to induce in IgA^+^ memory B cells (50) and that measles causes elimination of 11–73% of the antibody repertoire and depletion of previously expanded B memory clones after infection (51, 52). However, cellular and molecular mechanisms on their suppression are still unknown and should be investigated, then comparing with the case of *Salmonella*.

## Treatment of Antibody-Mediated Diseases Using a *Salmonella*-Derived Peptide

The SiiE peptide homologous to laminin β1 significantly reduced the number of anti-DNA IgG-secreting plasma cells in the BM in the NZB/W murine model of lupus nephritis (41). This property could therefore be further exploited for the treatment of autoimmune diseases. Autoimmune diseases with a substantial contribution of pathogenic IgG autoantibodies, like systemic lupus erythematosus, can be refractory to conventional treatment e.g., immunosuppressive drugs and anti-CD20 antibodies, because BM plasma cells secreting these autoantibodies are protected in their BM niches (53–55). Multiple myeloma is caused by redundant titers of antibodies generated from plasma cell myeloma in the BM. It has already been reported that myeloma cell lines preferentially contact laminin *in vitro* (56, 57), suggesting that targeting of adhesion molecules including laminin should be considered as novel therapy (58). The depletion of BM plasma cell myeloma by SiiE may directly ameliorate disease. SiiE peptide and the related products may contribute to a recovery for these antibody-mediated diseases without relapse.

## Conclusions and Perspectives

**Figure 2:**
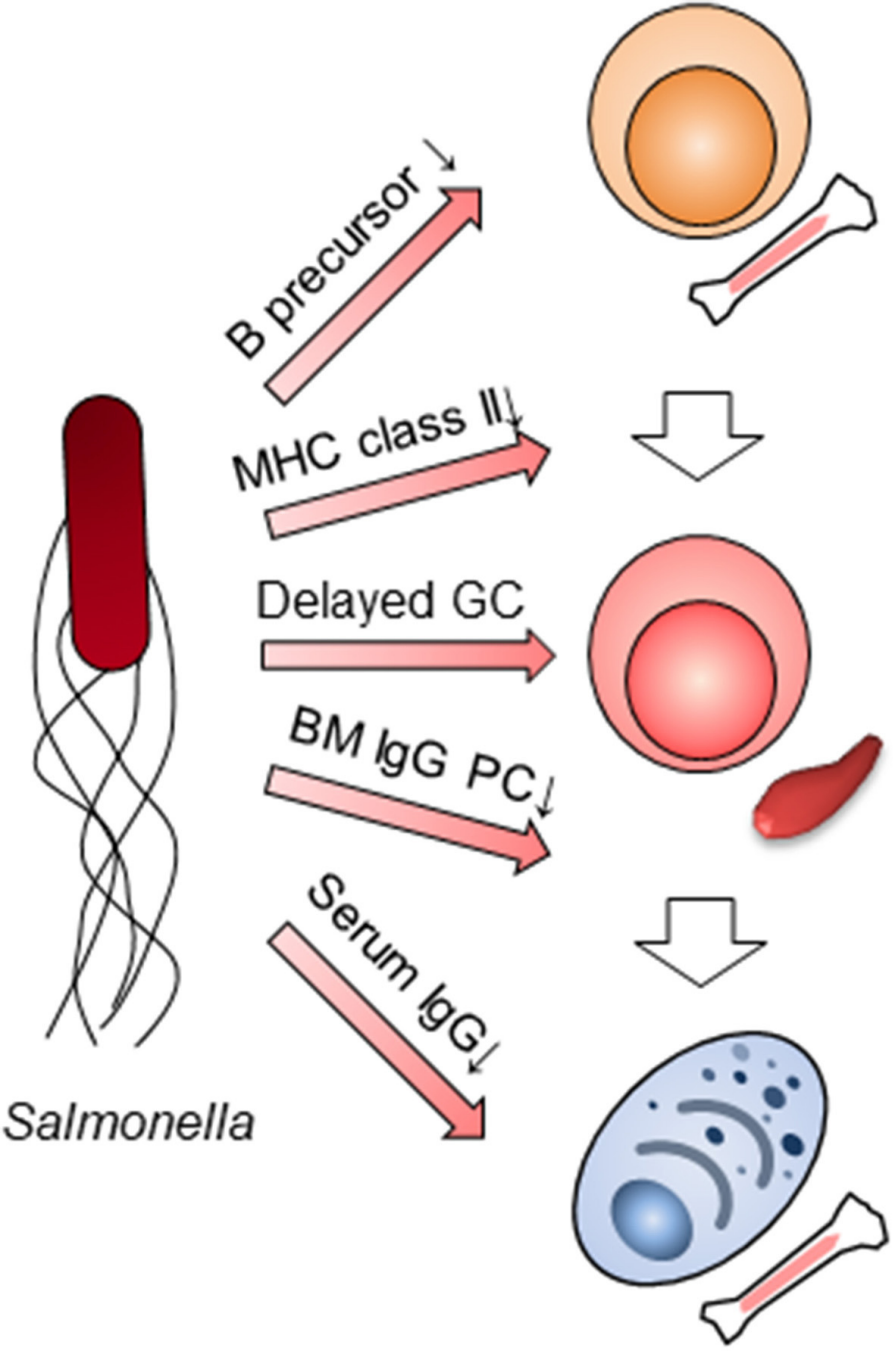
Multilayer suppression of humoral immunity by *Salmonella* infection. *Salmonella* impairs humoral immunity at multiple stages; B cell lymphopoiesis, the expression of MHC class II in myeloid cells, germinal center (GC) formation, the persistence of BM IgG-secreting plasma cells (PC) and IgG titers in serum.

Humoral immunity in the late phase of primary infection with *Salmonella* had been thought not to participate in the clearance of the bacteria. However, when taking into consideration *Salmonella*'s functions, it is clear that several aspects of humoral immunity, in particular the suppression of IgG production, does indeed contribute to the clearance of bacteria in the late phase of the primary infection (Figure 2). Using SiiE-deficient *Salmonella*, the collaboration between humoral immunity and other immune systems should be also re-evaluated. The function of other immune cells may be overestimated or underestimated due to the suppression of humoral immunity. Furthermore, the previous candidates of vaccines against NTS should be re-investigated by adding a mutation of SiiE. The combined mutations of *Salmonella* factors which interfere with immune systems may result in the development of the best vaccines against NTS. As infection with NTS may delete all IgG plasma cell memory gained by vaccination obtained from infancy, we therefore also alert to this danger and propose an obligatory use of vaccination against NTS in infancy.

## Author Contributions

AT and KT wrote the paper. TY supervised the work.

### Conflict of Interest

The authors declare that the research was conducted in the absence of any commercial or financial relationships that could be construed as a potential conflict of interest.
